# Dark Triad Traits Affect the Perception of Emotions in Animal Calls

**DOI:** 10.1002/ijop.70205

**Published:** 2026-04-03

**Authors:** Iva Linda Maruščáková, Lea Jakob, Hana Vostrá Vydrová, Marek Špinka

**Affiliations:** ^1^ Department of Psychology, Faculty of Arts Charles University Prague Czechia; ^2^ University of New York in Prague Prague Czechia; ^3^ Department of Ethology and Companion Animal Science, Faculty of Agrobiology, Food and Natural Resources Czech University of Life Sciences Prague Czechia

**Keywords:** animal emotion recognition, animal vocalisations, dark triad, emotion perception, personality

## Abstract

Humans can recognize emotions from vocalisations of various animal species. Our study examined whether human psychological differences in dark personality traits (as measured by SD3) and musician experience affect the decoding of emotions in animal calls. Respondents assessed the situation and the valence and intensity of emotion experienced by the animal in calls of piglets recorded in three social and one painful situation. With increasing psychopathy scores, individuals made more misclassification errors between social and painful calls and also perceived the social calls as more negative. Higher Machiavellianism scores were associated with a more positively perceived valence of social and painful calls. Furthermore, respondents with musician experience and using Czech (as opposed to English) positively shifted the perceived valence of social calls. These findings indicate that humans with higher psychopathic traits may possess mechanisms that blunt the difference between distressing and positive vocal signals, thus making it easier to exploit or manipulate others. Furthermore, interindividual personality differences and musical experiences influence how humans perceive emotions in vocal signals devoid of verbal cues. The implications are made for human‐animal interaction, the general dark triad theory, and the perception of emotions in nonverbal human infant calls.

## 1 Introduction

### 1.1 Animal Emotions in Psychology

The modern science of communication and understanding of animals' emotions was founded on observations made by Darwin after the long Cartesian denying of the capacity of animals to even feel and communicate. Darwin stated that emotional expressions are universal across mammalian species. A century later, this concept was developed into the theory of motivation‐structural rules theory about bird and mammalian sounds (Morton 1977). The theory posits an almost universal pattern in relation between the motivation or emotional state of the sender (e.g., aggressive, fearful, or friendly) and the acoustic quality of vocalisations emitted in such situations (Morton 1977).

In psychology, we do not have one commonly accepted definition or system for the classification of emotions, neither for humans nor nonhuman animals (hereafter animals). Most studies focus on the two most widely used approaches. One assumes that discrete, modular emotional states exist both in humans and animals that are a basis for emotion primitivés (French for primary emotions) upon which more complex, subjective feelings specific to humans such as grief, despair, or shame are built. The other approach is a dimensional circumplex model that postulates emotions as affective experiences varying in valence (pleasant or unpleasant) and arousal (high or low). This approach is useful when we are unsure about the exact quality of emotion of the subject being evaluated, as is often the case in animal emotions. For this reason, we used this approach in our study.

Emotions have their social aspect as they are expressed through various communicative means such as body postures, facial expressions, and vocalisations, and these signals can be recognized by both conspecifics and individuals of other species. For instance, non‐experts or veterinary practitioners can visually recognize the emotions of horses or cats from body postures or facial expressions. Besides visual decoding of expressed emotions, sounds are another communicative aspect of animal emotions. Darwin's postulate implies that humans can, to some extent, decode emotions across species (or at least across mammalian species) universally because they ‘sound’ similarly. Many species' affective vocal repertoire is now well mapped, including, for instance, pigs (Tallet et al. 2013).

### 1.2 Personality Factors and the Recognition of Emotions

In human emotional communication, a strong ability to recognize emotions is often connected to concepts such as higher emotional intelligence. Some authors consider emotional recognition as a part of Interpersonal accuracy, a construct focusing more narrowly on recognizing emotions, personality, intentions, motives, and thoughts compared to the broader concept of emotional intelligence that also covers emotional regulation skills. Personality traits of the Big Five concept, including Neuroticism, Agreeableness, or Extraversion, seem to have effects on the emotional recognition of others too.

Surprisingly, very little is known about whether there exist any individual psychological differences or skills among human listeners that might influence their ability to decode emotions in animal calls. Only a few studies so far have focused on psychological variables that might be crucial when we ask if and why some people understand animals' emotions in their calls better than others. In our previous study, we examined whether the Big Five Personality Traits or measures of empathy such as the Interpersonal Reactivity Index affect the ability to assess emotions in calls of pigs and found no such effect.

### 1.3 Dark Triad Traits and Emotional Recognition

In this study, we examine the influence of the dark triad traits of personality, measured with the Short Dark Triad (SD3; 2014) as defined by Jones and Paulhus (2014). The dark triad concept is an umbrella term for persons scoring higher on the three socially aversive personality traits consisting of narcissism, Machiavellianism, and psychopathy. This concept serves as a non‐clinical measurement for the “healthy” population and is often also connected to different processing of emotions of others, at least in human‐human interactions. The process of managing others' emotions might be either used in a positive way—for correct judging and positive reaction to the emotions of other people who feel, for example, sad—or in a negative way for their self‐serving purposes, for example, to make others behave in a way the person wants, or to induce desired negative feelings in them. Accordingly, the ability of emotional recognition could be either positively or negatively related to SD3. Recent meta‐analyses concluded that psychopathy and Machiavellianism traits tend to correlate negatively with emotional recognition of other humans while the narcissistic trait shows either no or weak positive correlation (Michels and Schulze 2021). The debate on this topic is ongoing and a detailed look at the three dark triad traits shows a mixed picture.

Although the three Dark Triad traits are moderately correlated and share features such as low empathy and callous affect, they also have important distinctions in emotional, motivational, and cognitive functioning (Jones and Paulhus 2014). For example, psychopathy is more strongly associated with affective deficits and impulsivity, Machiavellianism with strategic manipulation and emotional detachment, and narcissism with egocentric self‐enhancement. These differences can have different effects on emotional perception, such as sensitivity to emotional valence or accuracy in categorisation tasks. In this study, we treat the three traits as distinct dimensions, enabling us to examine potential shared versus unique effects. Although we hypothesize that all three traits may influence emotional perception, this analysis is partly exploratory, given the mixed findings in previous literature. Therefore, we interpret both common patterns and trait‐specific effects in our analyses and discussion.

#### 1.3.1 Psychopathy Trait

The level of psychopathy has been considered a significant factor causing the differences between persons' ability to assess human emotional content (Blair 2008; Walker et al. 2022). In several studies, persons scoring high on psychopathy were proven as being less sensitive to the emotions of others due to lower activation of the amygdala and the insular and orbitofrontal cortex having the lower ability to take the perspective of others and thus experiencing lower empathy levels, having lower capacity to recognize human distress cues, including sadness and fear or failing to distinguish between some vocal cues as in case of dominant and affective human laughter (e.g., Ali et al. 2009). The possible explanation of this compromised emotional recognition is that it helps psychopathic persons manipulate others without getting ‘disturbed’ by the expressions of their negative feelings.

On the contrary, other studies indicate that some people scoring high on the psychopathy scale may excel at processing the emotions of others in order to manipulate them. Thus, psychopathic traits may be connected with an improved capacity to assess the vulnerability of victims only from their body language or to recognize fearful facial expressions (Hastings et al. 2008). The picture is complicated by the possibility of distinguishing between primary psychopathy characterized by cruelty, lack of affect, and inability to experience negative emotions, often believed to be more biologically driven and secondary psychopathy linked with impulsivity, irresponsibility, neuroticism, and aggression, often related to early environmental risk factors such as trauma (Moreira et al. 2020). Primary and secondary psychopathy traits may affect emotional recognition differently, for example, when identifying fearful facial expressions (Hastings et al. 2008).

Beyond influencing emotional recognition, psychopathy may also shift the perception of emotional intensity and/or valence. For instance, positive, neutral, and negative human‐like stimuli may be perceived differently by people varying in either primary or secondary psychopathy (Ali et al. 2009).

#### 1.3.2 Machiavellism Trait

Machiavellian trait description originates from the philosopher Machiavelli and is characterized by presenting a cynical worldview, callous affect, lack of morality, manipulativeness, and domineering. What distinguishes Machiavelli's characteristics from psychopathy traits is planning, strategy setting, and supporting the best reputation in a social environment. People high on Machiavellian also avoid manipulating close persons and family.

According to some studies, a relationship between Machiavellian traits and deficiency in emotion recognition exists and people scoring high on this trait may exhibit difficulties in attributing complex emotions to others. Machiavellianism has been reported to correlate negatively with both cognitive and affective empathy, that is connected with emotional recognition as well (Al Aïn et al. 2013; Ali et al. 2009).

Finally, Machiavellianism may also have the effect of shifting the perceived intensity and/or valence of emotional stimuli. For instance, Machiavellianism was associated with experiencing positive affect from sad stimuli and negative affect in response to neutral, ambiguous stimuli (Ali et al. 2009). Some studies also confirm a positive association between Machiavellianism and levels of alexithymia, anhedonia, depression, and anxiety (Al Aïn et al. 2013).

Machiavellianism may influence valence perception through several interrelated psychological mechanisms. Individuals high in Machiavellian traits often exhibit emotional detachment and alexithymic tendencies, which can blunt their sensitivity to emotional valence. Additionally, they tend to engage in cognitively driven emotion regulation strategies, reinterpreting affective stimuli using rational processes rather than affective resonance, which may alter their perception of emotional tone (Deak et al. 2017). Biases in social judgements also suggest that Machiavellians may be more likely to perceive negatively valenced actions as more blameworthy, and emotion recognition research indicates they may have specific deficits in decoding negative emotions. These findings together suggest that Machiavellianism may shape valence perception by dampening affective attunement and introducing cognitive distortions in interpreting emotional content.

#### 1.3.3 Narcissism Trait

Narcissism reflects a sense of dominance and superiority, and people who score higher on this trait tend to express excessive self‐importance and grandiosity. Narcissism seems to be the least correlating factor with components of emotional intelligence. Although some studies suggest that narcissism may impair recognition of specific emotions such as fear or disgust, recent studies report no consistent correlation between narcissism and emotional intelligence or emotional decoding ability. Similarly, no strong effect of narcissism on cognitive empathy has been found. Recent meta‐analysis shows that grandiose narcissism is weakly positively associated, while vulnerable narcissism is negatively associated with emotional intelligence, and these effects depend on the specific measures used (Nguyen et al. 2022).

However, some studies indicated that narcissism may reduce emotional recognition; current meta‐analyses found this trait not correlated with emotional intelligence or correlating with it weakly positively (Miao et al. 2019). The most recent studies, too, report no effect of narcissism on emotional decoding or cognitive empathy.

### 1.4 Other Factors in the Recognition of Emotions in Animals' Calls

Existing studies indicate that other psychological and non‐psychological factors such as gender, age, attitude towards or experience with animals, musicality, or cultural background might influence how people perceive and interpret the emotional content of animal sounds (Tallet et al. 2010). Therefore, we also examined them in the current study.

#### 1.4.1 Attitudes Towards Animals and Experience With the Animal Species

The success of human emotional decoding may depend not only on the ability to recognize emotions from their expressions but also on the attitudes towards the task and the motivation to succeed in it. In our previous study on emotional recognition in animal voices, we examined the role of attitudes towards animals by using the Animal Attitudes Scale, with no effect found. As this self‐report measure is vulnerable to self‐biased judgements, we decided to use a direct behavioural form of expressing attitudes towards animals in the current study. Based on the Meat‐related cognitive dissonance concept, the choice of not consuming animals might be an example of such direct behavioural expression, at least for people choosing to do so for ethical reasons (Rothgerber 2020).

Furthermore, decoding emotions from animal calls may partly depend on the listener's experience with the particular animal species, since the vocal repertoires are species‐specific (e.g., Tallet et al. 2013). Indeed, professional pig ethologists were better than people with little pig contact at recognizing situations in which piglet calls were recorded while pig caretakers evaluated the intensity of emotions in the calls as lower, possibly as a ‘hardening or detachment’ coping mechanisms—denial or even dissociation in order to cope with the everyday exposure to negative pig vocalisations (Tallet et al. 2010). Therefore, we examined the experience with pigs as a possible factor affecting emotional recognition in piglet calls also in this study.

#### 1.4.2 Basic Sound Characteristics and Musical Experience

There is evidence to suggest that bioacoustics characteristics of calls might be among the cues on which vocal emotion recognition is based. Pongrácz et al. (2005) https://paperpile.com/c/h0uUuw/7be2V reported that human‐judged emotionality of dog barking correlated with peak and fundamental frequency as well as inter‐bark intervals. In piglet calls, vocalisations were rated by listeners as more intense with increasing pitch (mean fundamental frequency) and with increasing proportion of vocalized sound within each stimulus recording and more negative with increasing pitch and increasing duration of the calls within the recording. Musical training can enhance the accurate perception of acoustic features in vocally expressed emotions (Strait et al. 2009). Therefore, we added human musical experience as another factor in this study.

To date, little is known about how socially aversive personality traits, encompassed by the Dark Triad, shape individuals' ability to perceive or evaluate emotional signals in nonhuman animals. While some research (as mentioned above) has explored the effects of empathy and Big Five traits on emotion recognition, findings have been inconsistent, and few studies have examined how darker personality dimensions, such as those in the Dark Triad, may influence this perceptual process. Given the role of the Dark Triad traits in emotional detachment, manipulation, and altered empathy of the individual, they could uniquely affect how emotional content in animal vocalisations is recognized or appraised. As this topic remains under‐explored, the present study aims to address this by exploring how the Dark Triad traits relate to both the accuracy of emotional recognition and the subjective evaluation of intensity and valence in piglet vocalisations.

## 2 Aim and Hypotheses

The aim of the study was to examine the influence of SD3 traits on the recognition and perception of emotions in animal calls recorded in differently valent situations. Specifically, we tested two hypotheses:

Hypothesis H1 *The S*D3 *traits will affect the ability to recognize differently valent situations from animal vocalizations*.

Hypothesis H2 *The S*D3 *traits will shift the perceived intensity and valence in animal vocalizations*.

Besides, we also examined the effects of other personal factors including age, gender, experience with pigs, not/eating meat, and musical experience on the perception of animal calls.

## 3 Methods

### 3.1 Participants

Responses to the questionnaires (see below) were collected through an online snowball sampling method. Adults capable of reading and understanding Czech or English were invited to participate in the online study. Social media sites, such as Facebook (including specialised groups), Reddit, and personal contacts were used to disseminate the questionnaire link to interested participants.

The whole questionnaire was completed by 154 participants, of which 62 participants used the Czech version of the questionnaire and 92 the English version. The majority of participants were women (77.9% women, 22.1% men), with an average age of 28 years (*M* = 28.00, sd = 9.09). Only 17 participants were parents, and 37.7% regularly played a musical instrument or sang. The majority have encountered a pig in real life at least once (97%), but only a few were often around pigs (2 individuals were in weekly contact).

Considering dietary habits, half of the participants followed a conventional diet (51.3%), 11.4% were flexitarian, 14.3% were vegetarian, and 23.4% were vegan.

### 3.2 Data Collection Design

The study employed completely anonymous and voluntarily filled online questionnaires. For playback stimuli, we used pre‐existing recordings that had been recorded in routine pig farm operations (Tallet et al. 2010). For these reasons, ethical review and approval were not required for this study according to Czech legislation and institutional requirements. Furthermore, the study was not preregistered.

The data collection proceeded online. First, participants were asked to provide informed consent before starting the questionnaires. Then, the data collection consisted of a personal questionnaire, a test on emotion assessment and situation recognition from pig vocalisations, and psychological questionnaires. The personal questionnaire gathered demographic data about the participants, including gender, age, experience with interaction with pigs, eating or not eating meat/animal products, and whether the person actively performs music by singing or playing an instrument.

### 3.3 Measures

#### 3.3.1 Emotion Assessment and Situation Recognition in Pig Vocalisations

This study used 48 recordings of piglet vocalisations used in previous papers (Tallet et al. 2010). The sounds were recorded while 7–14 days old piglets were in one of four situations, namely post‐suckling, reunion with the mother sow, brief isolation, and castration which is performed routinely in male piglets on most European farms (Council Directive 2008/120/EC 2008). Each of the 48 sounds was recorded from a different piglet. For more details on the stimuli, see Tallet et al. (2010). Each of the four sound categories included 12 sound examples from which randomly selected playback was drawn. The sounds were used in three tasks. For each participant across the three tasks, 36 out of the 48 sounds were used. The specific set of 36 sounds was randomly drawn and therefore different for each participant.

The first task was to listen to a randomized vocalisation and determine the level of perceived intensity (Likert scale 1 to 5, 1 = *Very low* to 5 = *Very high*) of the emotion the animal may be expressing. This task was done 12 times, with three sounds from each of the four categories being presented in a random order. In the second task, the participant was asked to estimate the valence of 12 different randomly selected recordings by indicating the perceived level of satisfaction or dissatisfaction the pig was expressing on a 5‐point Likert scale (1 = *Strong dissatisfaction* to 5 = *Strong satisfaction*). During these two tasks, the participant was not given any information about the situations in which the sounds were recorded. Lastly, the participants were asked to listen to a vocalisation sample and choose one of the four potential situations (post‐suckling, reunion with the sow, isolation, and castration) in which the sound may have been recorded. For this last task, yet another set of 12 randomly drawn vocalisations (three per each situation) from the pool of 48 recordings was used. As the three sets of sounds were drawn randomly, the three sets used in the three tasks for the same participant were different from each other. Yet, due to the random drawing, sometimes particular sounds occurred randomly in two or even in all three sets for the same participant.

#### 3.3.2 Measures of Dark Personality Traits (SD3)

In the third part of the data collection, three psychological questionnaires were administered: The Defence Style questionnaire, The Short Dark Triad questionnaire, and the Learned Optimism Test, with only the Short Dark Triad questionnaire data being reported in this paper.

The Jones and Paulhus' (The Short Dark Triad; 2014 https:// paperpile.com/c/h0uUuw/YeePO/?noauthor=1) questionnaire was used to measure the personality traits that correspond to the dark triad personality model. This model postulates that there are three traits—Machiavellianism, psychopathy, and narcissism—which represent non‐clinical personality qualities related to negative social outcomes and detrimental behaviours.

The items are answered on a 5‐point Likert scale (1 = *Disagree strongly* to 5 = *Agree strongly*). For this study, we used the English and Czech versions of the questionnaire. The Czech version was previously validated by Mejzlíková et al. (2018).

### 3.4 Data Analysis

Statistical evaluation started with descriptive quantification of the recognition rate, perceived intensity, and perceived valence of the calls recorded in the four situations. This analysis showed that nursing, reunion, and isolation calls were perceived similarly while castration calls were perceived as clearly different. Therefore, the testing of the hypotheses proceeded separately for castration calls and separately for the pooled category of social calls which included calls from the remaining three situations.

To test Hypothesis H1 about the influence of the three dark triad traits on recognition of the correct call category (castration or social), a generalized linear model (*proc glimmix* in SAS 9.4) with Poisson distribution was run. In the model, the dependent variable was the number of categorisation errors (with possible range of errors between 0 and 12 and the actual number of errors ranging between 0 and 5 out of 12 possible) and the fixed factors were the psychopathy, narcissism, and Machiavellianism scores of participants, the demographic parameters of age, gender (female or male), and language (Czech or English) as well as the individual parameters of experience with pigs, musician experience, and dietary habit. General linear models were employed to test Hypothesis H2 about the influence of the dark triad on perceived intensity and valence. The dependent variables in the models were the average intensity and average valence assigned to the 12 calls by the individual participant, and the fixed factors were the same as those in the above model on recognition. The model on intensity and valence was run separately for the castration and social calls. Three participants were omitted from the analysis due to incomplete data. Two participants who provided stereotyped judgements across all vocalisations were also disregarded, thus reducing the final sample size to 154 participants. Parenthood was not included in the models as only 10% of the respondents were parents. The models were checked for normality of residuals and homoscedasticity using scatterplots of residuals versus predicted values.

## 4 Results

### 4.1 Descriptive Statistics

Considering the four different situations in which the sounds were presented, the participants provided their judgement (1) in which situation the vocalisation was recorded, (2) what is the subjectively perceived intensity of the emotion in the call and (3) what is the subjective perception of the emotional valence. The descriptive analysis (Table 1) indicates that calls emitted during castration were different from the other three situations: they were most easily recognized and judged to be of much higher intensity and more negative valence. The other three situations had a similar recognition rate and were evaluated similarly in terms of their intensity and valence. Therefore, the hypotheses testing was accomplished separately for castration calls and the pooled category of social calls (nursing, reunion, and isolation).

**TABLE 1:** Mean (and standard deviation) of different vocalisation type perceptions based on the situation (*N* = 154).

	Nursing	Reunion	Isolation	Castration
Average correct vocalisation‐situation recognition	47.1% (28.7%)	42.3% (28.5%)	32.0% (26.3%)	82.3% (24.7%)
Perceived intensity of emotion	2.73 (0.57)	2.83 (0.61)	2.72 (0.61)	4.63 (0.42)
Perceived valence of emotion	3.11 (0.59)	3.75 (0.53)	3.09 (0.58)	1.34 (0.42)

### 4.2 Hypothesis H1: Effects of Dark Triad on Situation Recognition

The recognition of whether the call originated from the castration or a social situation was high overall (91%). Still, the recognition of the situation was affected by psychopathy, thus partly confirming Hypothesis H1 (Table S1). Specifically, respondents with higher psychopathy scores committed more misclassification errors between castration and social calls (*F* (1,144) = 5.27, *p* = 0.023). The other two dark triad traits did not affect the situation recognition.

### 4.3 Hypothesis H2: Effects of Dark Triad on Intensity and Valence Perception

**FIGURE 1:**
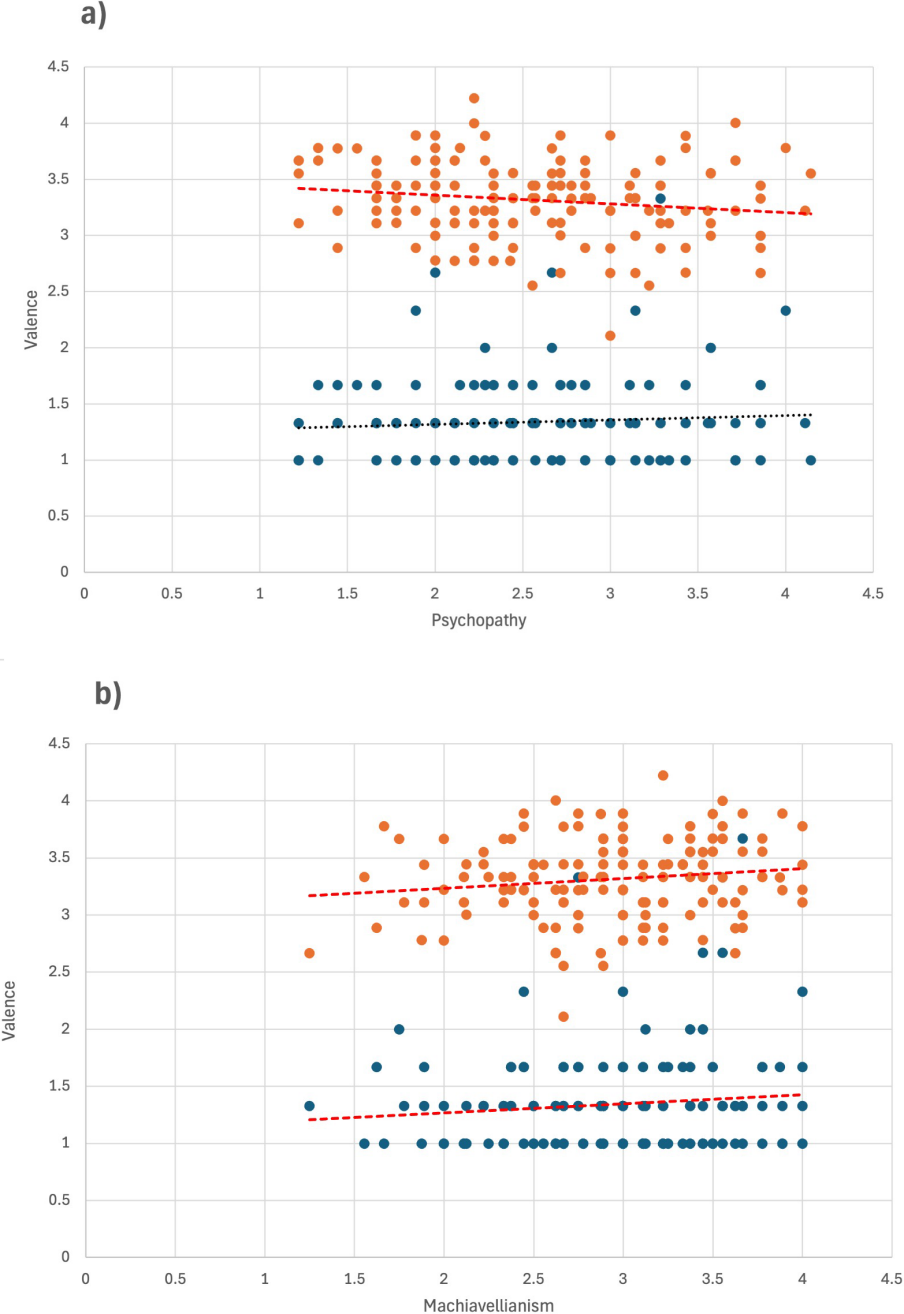
(a) The influence of psychopathy on estimated valence for castration (blue dots) and social calls (orange dots). (b) The influence of Machiavellianism on estimated valence for castration (blue dots) and social calls (orange dots). Red fitted lines are for significant effects, the black fitted line is for non‐significant effect.

The perceived intensity of the calls was unaffected by the dark triad traits (Table S2). In contrast, the perceived valence of the calls was influenced by two dark triad traits (Table 2, Figure 1). People with higher psychopathy scores had lower perceptions of valence in social calls, that is, they perceived them as more negative. Higher Machiavellianism scores were associated with higher perceived valence in both castration and social calls, that is, they perceived them as more positive.

**TABLE 2:** Effects of dark triad traits on the perceived valence in piglet calls.

Effect	Estimate	*F*	df	*p*
*Castration calls*				
Psychopathy	−0.026	0.12	1, 144	0.729
Machiavellianism	0.156	4.34	1, 144	0.039
Narcissism	−0.113	2.73	1, 144	0.101
*Social calls*				
Psychopathy	−0.195	9.99	1, 144	0.002
Machiavellianism	0.186	9.68	1, 144	0.002
Narcissism	−0.062	1.3	1, 144	0.257

### 4.4 Effects of Other Factors

**FIGURE 2:**
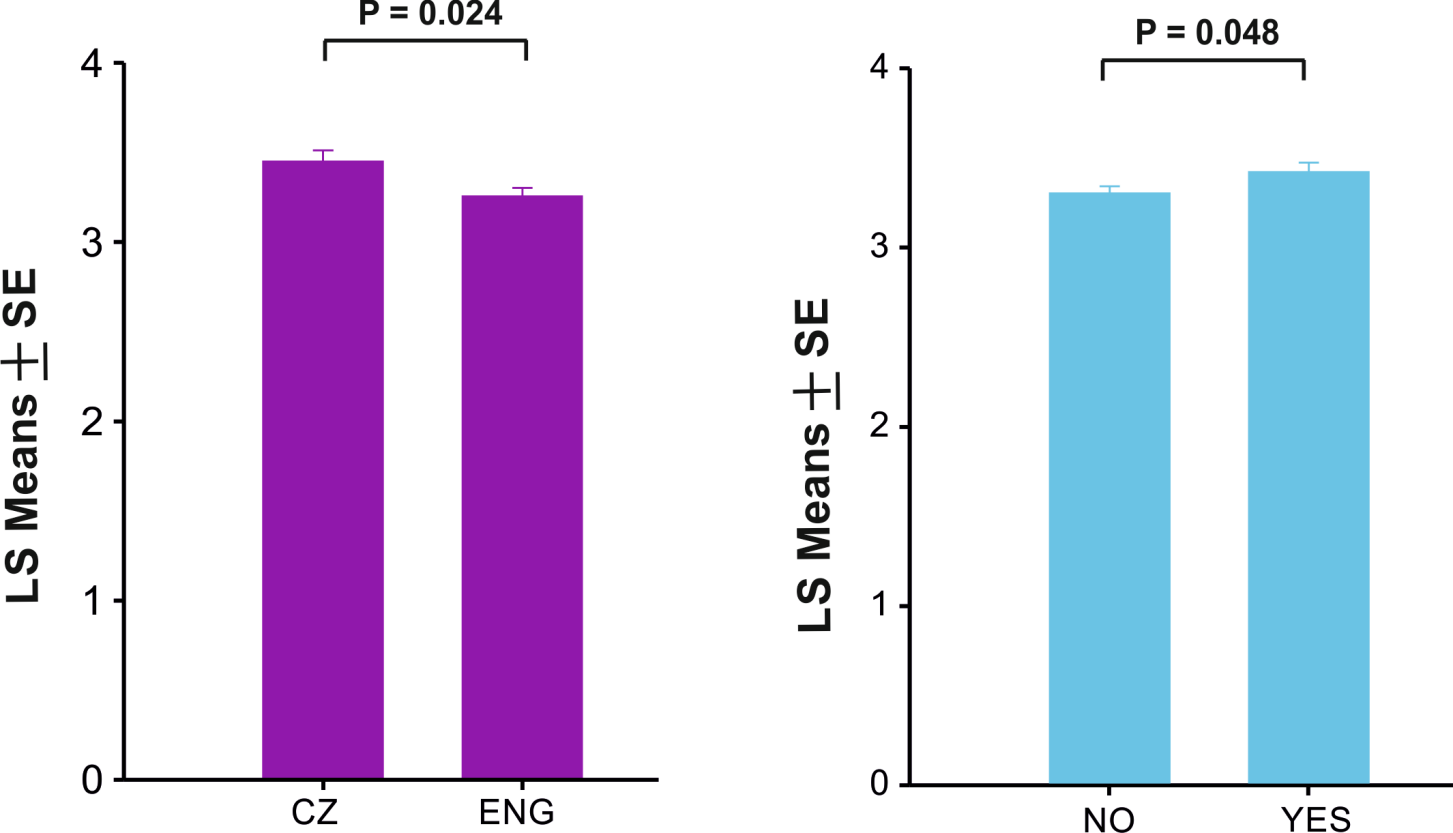
The difference in valence perception between individuals who completed the testing in Czech and English language (left graph) and who had musical experience (right graph).

The examined demographic and individual variables did not influence situation recognition or intensity perception (Tables S1 and S2). However, perception of valence was affected by language and musician experience (Figure 2). The valence of the social calls was perceived as higher (more positive) by participants using the Czech questionnaire than by participants using the English version (*F*(1,144) = 5.20, *p* = 0.024). Also, people with musician experience perceived social calls as more positive (*F*(1,144) = 4.00, *p* = 0.048).

## 5 Discussion

This is the first study to examine how dark triad traits influence the recognition and perception of emotions expressed in animal voices. We examined two hypotheses: first, that the dark triad traits influence the ability to recognize animal calls recorded in differently valent situations (painful experience versus social interaction), and second, that the same factors cause a shift in the perceived emotional intensity and valence from the calls.

As there are no studies linking dark triad personality traits to recognition or perception of animal emotional cues, we discuss the current results mainly in relation to previous research in which human, rather than animal, emotional signals were used. However, even this research is not balanced. While numerous investigators studied the effects of psychopathy (variously conceptualized and measured) on recognition of human cues representing differently valent situations, the impact on perception shift, that is, whether psychopathy increases or decreases the perceived intensity or valence of the cues, was less frequently examined. Also, most studies used visual stimuli (face or body images or videos) rather than vocal cues. Finally, the influence of the Machiavellian and narcissistic traits either on emotional recognition or on shifts in emotional perception were investigated less frequently than the psychopathy effects.

In the current study, we found just one effect related to the recognition Hypothesis (H1) while several effects were related to the perception shift Hypothesis (H2). In a similar vein, Wai and Tiliopoulos (2012) reported little evidence that dark triad personality traits were associated with cognitive empathy (the ability to discern the emotional states of others) while affective empathy (i.e., emotional reaction to others' emotions) was more strongly affected by the dark triad traits.

### 5.1 Hypothesis H1: Effects of Dark Triad on Situation Recognition

Our results indicate that persons who score higher on psychopathic personality traits do worse on the situation‐sound matching test, thus partly supporting H1. Specifically, with increasing non‐clinical psychopathy scores, the probability increased that the individual would make more mistakes in the classification of the calls to the social versus the castration situation.

Existing studies from human psychology are not in accordance with whether psychopathic traits are associated with better or worse emotion decoding, with several studies reporting deficits in the recognition of negative emotions, particularly fear and sadness (Hastings et al. 2008).

However, the effect of psychopathy on categorisation performance appeared trait‐specific, as similar associations were not observed for Machiavellianism or narcissism. This aligns with prior findings suggesting that psychopathy is uniquely associated with deficits in affective empathy and processing of distress cues (Blair 2008), which may directly impair the recognition of negatively valenced or socially salient vocalisations. These results support the notion that emotional decoding deficits are more strongly tied to psychopathy than to the other Dark Triad traits.

Both approaches suggest the same reason for such a mechanism, namely, that it helps psychopathic persons to better cope with possible (visual or vocal) cues of the suffering of victims or, in the case of humans, manipulated and misused persons. Our finding rather supports the prevailing view that psychopathic persons are less able to recognize distress signals across modalities, including sad, fearful, painful, or angry vocal cues or fearful facial expressions (Montagne et al. 2005).

Our results suggest that persons scoring high on the psychopathy scale might use the “predator” mechanism of worse recognition of pain vocal signals also across species, which is an interesting fact, perhaps confirming the same evolutionary basis of perceiving the distress vocal signals. However, as this was the first study of its kind, further research is essential. Especially, it would be interesting to examine how the primary or secondary psychopathy or facets of psychopathy (e.g., effective‐interpersonal versus lifestyle‐antisocial, callousness versus meanness) influence the recognition of animal emotions from animal calls as these components of psychopathy affect differently the decoding of human emotional cues (Ali et al. 2009; Cigna et al. 2017).

### 5.2 Hypothesis H2: Effects of Dark Triad on Perceived Valence

None of the three SD3 traits affected the respondents' judgement of the intensity of emotions contained in the calls. On the other hand, the judged valence was affected by the psychopathy and Machiavellianism trait scores, thus partly supporting H2. In agreement, Wertag et al. (2024) found that dark triad traits were related only to valence, but not to arousal ratings of visual affective stimuli.

We found that with increasing non‐clinical psychopathy scores, the social calls were assigned lower valence, that is, they were perceived as less positive. Also, with increasing Machiavellianism scores, both categories of calls were judged to have higher valence. That is, more machiavellian persons perceived the castration calls as less negative and the social calls as more positive. Thus, psychopathy and Machiavellianism seem to have, according to our results, different effects on the perception of emotions from animal calls, while narcissism does not influence the perception.

The fact that psychopathy scores had an influence on the valence perception of social calls (which were generally perceived as neutral or mildly positive, see Table 1) and not on the strongly negatively perceived castration calls is intriguing. Together, these two results may indicate that the valence perception of animal sounds is flattened in persons with higher psychopathy scores. Specifically in relation to animal vocalisations, limiting the perceived positivity of vocal cues might help persons leaning to psychopathy to shield off, to some extent, perceiving the emotions that across species serve as a signal of internal states. And to psychopaths, this might serve also as a way to reduce their sensitivity to other beings.

The increase of valence perception in both call categories with Machiavellianism may suggest that persons scoring high in this trait are inclined to shift their judgement about the experienced emotion of the animals in the positive direction.

It is not easy to relate our results on perceived valence to previous research. In comparison with the numerous studies on psychopathy's effects on emotional recognition (see discussion above) and also on psychopathy's influence on neural responses, only a few studies examined the effects of psychopathy on shifts in verbally reported valence judgements of emotional cues and all of these studies are limited to visual stimuli. Truedsson et al. (2019) reported that young humans with higher callous‐unemotional traits (a factor in psychopathy) assign less positive valence to positive affective images which partly corroborates our finding. However, Deming et al. (2022) found no effect of psychopathy on the perceived intensity or valence of static or dynamic emotional visual stimuli.

As for the effects of Machiavellianism, the available literature is even more sparse. Wertag et al. (2024) recently found that higher Machiavellianism was associated with shorter response latencies in the rating of positive affective pictures, but the valence ratings of either negative or positive images were unrelated to Machiavellianism.

The observed association between Machiavellianism and altered perception of emotional valence may be due to emotional disengagement and reappraisal biases in individuals high on this trait. Individuals who score high on this trait tend to employ cognitive emotion regulation strategies, such as suppression and reinterpretation, which may blunt affective resonance and shift perception towards more detached, neutral, or strategic interpretations of emotional content (Deak et al. 2017). Previous studies have shown that Machiavellian individuals may misattribute negative valence to ambiguous or neutral stimuli (Ali et al. 2009), possibly related to the tendency to assume a cynical worldview or default mistrust. This may explain the increased negativity bias or reduced sensitivity to emotional nuance we have observed. Thus, while Machiavellianism may not impair emotion recognition per se, it may shape how emotional signals are subjectively evaluated.

In future research, it might be interesting to link these results with the existing findings on the discrimination of basic auditory features in human auditory system. such as pitch, loudness, frequency composition and salience. Features such as pitch, intensity, and salience of natural sounds including animal calls are coded in the antero‐lateral Heschl's gyrus (HG) and adjacent superior temporal gyrus (STG) of the auditory cortex, and the perception of these features differs between individuals (Jaatinen et al. 2021). Animal calls emitted in various situations and affective states differ in their acoustic quality (Tallet et al. 2013), and differing perception of the acoustic features by humans varying in their psychological profiles may be one of the mechanisms the effects observed in the current study.

Extending beyond the current focus on the three dark triad traits, prior research has found that Big Five personality traits and empathy measures show inconsistent associations with emotion recognition in animal vocalisations while here we show several Dark Triad traits to be predictive of participants' perceptual responses. One explanation is that the motivational orientation and social cognition deficits characteristic of the Dark Triad may more directly influence how emotional signals are interpreted and valued, rather than simply recognized. For example, psychopathy and Machiavellianism may shape the evaluation of emotional stimuli by modulating affective resonance, threat sensitivity, or strategic reasoning. On the other hand, Big Five traits' influence on social behaviour is less specific and may not consistently translate into perceptual differences in affective decoding. This suggests that the Dark Triad could be capturing a distinct psychological domain more relevant to interpreting emotional cues that are considered ambiguous or based on biological contexts such as animal vocalisations.

### 5.3 Language Affects the Valence Judgement in Social Calls

The valence of the social calls was perceived more positively by participants of the Czech questionnaire than by participants using the English version. One of the possible explanations for the finding can be due to prosodic phrasing, as the Czech language has a flatter intonation while English speakers are more exposed to dynamic pitch changes in the language (Skarnitzl and Hledíková 2022). This difference in speech melody may affect how speakers of each language assess the emotional content of animal calls as the moderate frequency modulation typical for piglet social calls (Tallet et al. 2013) could be perceived as more melodical compared to their spoken language for native Czech speakers than native English speakers. Nevertheless, Czech and English speakers likely differ, on average, in a number of culturally influenced aspects of auditory perception, emotional appraisal and cognitive attitudes.

### 5.4 Musical Experience Shifts the Valence Judgement in Social Calls

We found that increased experience with music shifted the perceived valence of social calls in a positive direction. Engaging with music through listening or performing has multifaceted effects on human behaviour, cognition, and affective processing. Specifically, musical training promotes the perception of vocally expressed emotions, possibly through enhancing subcortical auditory processing of the spectrally most complex portions of human affective vocalisations (Strait et al. 2009). Increased musicality, be it through training, interest, or intervention, makes humans more skillful in vocal emotion perception. This enhanced skill may be mediated by the musicians' enhanced ability to follow the melody and/or the rhythm of vocal emotions (van der Aa and Fitch 2025 https://paperpile.com/c/h0uUuw/4yr0). Also, expert music listeners attend to more complex features in music. Our previous study documented that the acoustic qualities of the pig calls are decisive for the perception of their valence. Therefore, listeners with music experience may have found a richer sound quality in the social calls of pigs, and therefore they perceived them as more positive.

### 5.5 Limitations

Due to the snowball recruitment method, our sample was not balanced. Specifically, young non‐parental women were overrepresented among the participants. This may have influenced the lack of gender effect and possibly also other results. Because we had quite a long testing battery, also containing other questionnaires on defence mechanisms and biases (results will be published separately), we used the Short Dark Triad questionnaire for the quantification of psychopathy, Machiavellianism, and narcissism. This tool does not distinguish between the various facets of psychopathy that are known to be differently related to emotional perception and recognition, and thus we could not study, for instance, what are the separate effects of primary versus secondary psychopathy.

### 5.6 Implications

This study shows that dark triad traits, and in particular psychopathy and Machiavellianism, affect the processing of young piglets' affective voices, including the calls produced in intense pain. This finding has implications in animal welfare where human perception, discernment, and reaction to animal affective vocal expression may affect how sensitive to animals' reactions are the persons responsible for animal care and what attitudes they have towards them. In addition, the finding implies that research should also address how dark personality traits impact the perception and evaluation of preverbal babies' vocal affective signals. Adult humans probably assess the affective content of preverbal children's calls through very similar mechanisms as they do for animal calls (Lindová et al. 2015), but the association between dark triad and perception of babies' affective calls has never been evaluated.

## 6 Conclusion

Despite its limitations, the current study is the first to examine the effects of dark triad traits on the recognition and perception of animal affective vocal stimuli. The findings suggest that in the adult non‐clinical population, higher psychopathy increases the error rate in discerning painful calls from social calls and that the individual differences in psychopathy and Machiavellianism have an influence on the valence rating of animal sounds. These results encourage further research into how persons differing in dark triad traits decode affective states from animal and human non‐verbal vocal cues.

## Author Contributions

**Iva Linda Maruščáková:** conceptualization, methodology, investigation, writing – original draft, writing – review and editing, project administration, funding acquisition, visualization. **Lea Jakob:** validation, writing – review and editing, data curation, formal analysis, methodology. **Hana Vostrá Vydrová:** software, data curation. **Marek Špinka:** supervision, formal analysis, writing – review and editing, validation, methodology, resources, software.

## Funding

The authors have nothing to report.

## Ethics Statement

The project was reviewed and approved by the Research Ethics Committee of Charles University. All participants involved provided informed consent, and the study was conducted in accordance with applicable ethical guidelines. This study utilized audio recordings obtained under the ethical approval granted for a prior research project (Tallet et al. 2010). All procedures involving animals were approved and conducted in accordance with Czech legislation on the protection of animals against cruelty.

## Conflicts of Interest

The authors declare no conflicts of interest.

## Supporting information


**Table S1:** Effects of Dark triad traits and other variables on misclassification errors between castration and social calls.

**Table S2:** Effects of Dark triad traits on the perceived intensity in piglet calls.

## Data Availability

We confirm that the data supporting the findings of this study are provided in the Supporting Information and in an open digital repository https://osf.io/mxef8/.
