# Supplementary material for: Dark Triad Traits Affect the Perception of Emotions in Animal Calls
Source: Int J Psychol. 2026 Apr 3;61(3):e70205. doi: 10.1002/ijop.70205 (PMC13049094; doi:10.1002/ijop.70205)
Supplement: Supplementary file 1 — Table S1: Effects of Dark triad traits and other variables on misclassification errors between castration and social calls. [file IJOP-61-e70205-s001.docx]

**Table S1**

*Effects of Dark triad traits and other variables on misclassification errors between castration and social calls.*

| **Effect** | **estimate** | ***F*** | ***df*** | ***p*** |
| --- | --- | --- | --- | --- |
| Psychopathy | 0.417 | 5.27 | 1, 144 | **0.023** |
| Machiavellianism | -0.128 | 0.53 | 1, 144 | 0.469 |
| Narcissism | -0.172 | 1.08 | 1, 144 | 0.301 |
| Language | -0.156 | 0.40 | 1, 144 | 0.531 |
| Age | -0.000 | 0.00 | 1, 144 | 0.933 |
| Gender | 0.057 | 0.08 | 1, 144 | 0.771 |
| Pig experience | -0.064 | 0.31 | 1, 144 | 0.576 |
| Diet | -0.104 | 2.15 | 1, 144 | 0.145 |
| Music | 0.217 | 1.37 | 1, 144 | 0.243 |
