# Supplementary material for: Dark Triad Traits Affect the Perception of Emotions in Animal Calls
Source: Int J Psychol. 2026 Apr 3;61(3):e70205. doi: 10.1002/ijop.70205 (PMC13049094; doi:10.1002/ijop.70205)
Supplement: Supplementary file 2 — Table S2: Effects of Dark triad traits on the perceived intensity in piglet calls. [file IJOP-61-e70205-s002.docx]

**Table S2**

*Effects of Dark triad traits on the perceived intensity in piglet calls*

| **Effect** | **estimate** | ***F*** | ***df*** | ***p*** |
| --- | --- | --- | --- | --- |
| ***Castration calls*** |  |  |  |  |
| Psychopathy | -0.003 | 0.00 | 1, 144 | 0.967 |
| Machiavellianism | 0.045 | 0.36 | 1, 144 | 0.547 |
| Narcissism | -0.042 | 0.37 | 1, 144 | 0.546 |
|  |  |  |  |  |
| ***Social calls*** |  |  |  |  |
| Psychopathy | 0.100 | 1.58 | 1, 144 | 0.210 |
| Machiavellianism | -0.066 | 0.72 | 1, 144 | 0.397 |
| Narcissism | -0.049 | 0.49 | 1, 144 | 0.485 |
